# High Whole-Genome Sequence Diversity of Human Papillomavirus Type 18 Isolates

**DOI:** 10.3390/v10020068

**Published:** 2018-02-07

**Authors:** Pascal van der Weele, Chris J.L.M. Meijer, Audrey J. King

**Affiliations:** 1 National Institute for Public Health and the Environment (RIVM), Centre for Infectious Disease Control, 3721 MA Bilthoven, The Netherlands; audrey.king@rivm.nl; 2 Department of Pathology, Vrije Universiteit—University Medical Center (VUmc), 1081 HV Amsterdam, The Netherlands; cjlm.meijer@vumc.nl

**Keywords:** HPV18, genetic epidemiology, whole genome sequencing

## Abstract

Background: The most commonly found human papillomavirus (HPV) types in cervical cancer are HPV16 and HPV18. Genome variants of these types have been associated with differential carcinogenic potential. To date, only a handful of studies have described HPV18 whole genome sequencing results. Here we describe HPV18 variant diversity and conservation of persistent infections in a longitudinal retrospective cohort study. Methods: Cervical self-samples were obtained annually over four years and genotyped on the SPF10-DEIA-LiPA_25_ platform. Clearing and persistent HPV18 positive infections were selected, amplified in two overlapping fragments, and sequenced using 32 sequence primers. Results: Complete viral genomes were obtained from 25 participants with persistent and 26 participants with clearing HPV18 infections, resulting in 52 unique HPV18 genomes. Sublineage A3 was predominant in this population. The consensus viral genome was completely conserved over time in persistent infections, with one exception, where different HPV18 variants were identified in follow-up samples. Conclusions: This study identified a diverse set of HPV18 variants. In persistent infections, the consensus viral genome is conserved. The identification of only one HPV18 infection with different major variants in follow-up implies that this is a potentially rare event. This dataset adds 52 HPV18 genome variants to Genbank, more than doubling the currently available HPV18 information resource, and all but one variant are unique additions.

## 1. Introduction

Persistent high-risk (hr) human papillomavirus (HPV) infection is the prime cause for cervical cancer [1]. Currently, 13 HPV types are considered to be high-risk based on criteria defined by the International Agency for Research on Cancer [2], with different carcinogenic risk for each hrHPV type. HPV16 and HPV18 cause approximately 70% of all cervical cancers worldwide [3], making them the primary target for research and vaccination alike.

HPVs are a strongly conserved dsDNA family of viruses. Individual HPV types are considered to show >90% sequence homology. HPV sequences with <10% nucleotide difference are considered the same HPV type, but can be subdivided into lineages (1–10% sequence difference) and sublineages (0.5–1.0% sequence difference). All sequences belonging to the same HPV type are considered variants [4]. Differential risks for cervical intraepithelial neoplasia (CIN) and cervical cancer have been analyzed for all hrHPV types [5,6,7,8]. A correlation between lineages and carcinogenicity has been described repeatedly for HPV16 [5,6,7,9] and conservation of HPV16 E7 has been associated with carcinogenesis [10]. For HPV18, three lineages and eight sublineages have been described (A1–5, B1–3 and C) [4]. However, unlike HPV16, some ambiguity exists in the current research on the carcinogenic properties of variant lineages for HPV18 [5,6,8]. For other hrHPV types, the literature on lineages and carcinogenicity is limited. Only one large study has been conducted describing some type-specific (TS) correlations [11]. The studies described have succeeded in establishing the importance of HPV variant identification in the context of persisting infections, CIN lesions and finally cervical cancer. Research on differences between persistent infections and clearing infections at the viral genome level is limited [11,12], especially for HPV18. High variant diversity has been described for HPV16 [9,13], but literature describing HPV18 diversity based on whole-genome sequencing (WGS) within populations is scarce [14,15,16]. Here we present the HPV18 variant diversity in a longitudinal study population in The Netherlands using a WGS approach. Additionally, we assessed the prevalence of HPV18 infections where a shift occurs in major variants after follow-up, which could be considered reinfection events and which would incorrectly be considered persistent infections based on conventional genotyping. These findings could be relevant in a context where vaccine efficacy and efficiency is determined [17,18].

## 2. Materials and Methods

### 2.1. Study Design

Samples were obtained from the *Chlamydia trachomatis* Screening and Implementation (CSI) study. This study was approved by the Medical Ethical Committee of VUmc Amsterdam (2007/239, METC VUmc, 2007) and registered in the Dutch Trial Registry (NTR3071, 16 September 2011). Recruitment criteria and methods have been previously described [19,20], along with additional consent required for HPV testing [21]. 3282 study participants consented to additional HPV testing and supplied samples in up to four round, with median fifty weeks between rounds (95% CI: 49–50 weeks, min: 5, max: 101 weeks). Ethnicity was based on country of birth and assigned according to Woestenberg et al. [22]. Participants were divided in European, Asian, African and “mixed” (Caribbean and Middle-American) ethnicities. Clearing infections were defined as type-specific HPV positive at the initial time point, followed by a type-specific HPV negative measurement. Persistent infections were type-specific HPV positive in at least two subsequent sampling rounds. Persistent infections were assessed for different major variants by sequence and phylogenetic comparison of initial and follow-up samples. If an infection was found to have different major variants at two subsequent time points, it was omitted from statistical (but not phylogenetic) analysis, since Sanger cannot reliably distinguish between persistent and clearing in these cases, due to limited sequence resolution.

### 2.2. DNA Purification and HPV Detection, Genotyping and Quantification

Total DNA was isolated from 200 µL of cervical self-swabs using the MagnaPure96 platform (Total Nucleic Acid Isolation Kit, Roche Diagnostics, Basel, Switzerland) according to the manufacturer’s protocol. Isolates were eluted in 100 µL and subsequently genotyped via the SPF10-DEIA-LiPA_25_ platform (DDL Diagnostics, Rijswijk, The Netherlands) [23,24]. HPV18 positive samples were quantified previously [25].

### 2.3. Sample Selection, Long-Template PCR and Sequencing

HPV18 positive samples with viral load >100 copies/µL (c/µL) were identified. Samples below this empirically defined threshold were likely to fail during amplification and therefore excluded. Persistent infections according to genotyping results were selected if the initial and last available follow-up samples were above the viral load criterium. In addition, 35 clearing HPV18 infections with VL above the arbitrary threshold were selected at random. Selected samples were subjected to an initial PCR amplifying the complete HPV18 genome in two overlapping fragments (F2458 + R6668 and F6538 + R3393 [15]). Amplification was performed using PrimeSTAR GXL DNA polymerase (Takara Bio, Kusatsu, Shiga, Japan). Cycling conditions consisted of an initial incubation at 98 °C for eight minutes followed by 38 cycles of alternating 98 °C for 15 s, 55 °C for 30 s and 68 °C for five minutes. A final elongation of 15 min at 68 °C was included. Amplification was verified on the FlashGel system (Lonza, Basel, Switzerland). Successfully generated amplicons were treated with ExoSAP-IT PCR product clean-up (Affymetrix, Santa Clara, CA, USA) according to manufacturer’s protocol. If the initial sample of a persistent infection failed to amplify, the follow-up sample was omitted from further analysis. If the initial sample succeeded, but the follow-up sample failed to amplify, these infections were included in phylogenetic analysis, but excluded from statistical and single-nucleotide polymorphism (SNP) analysis as true persistency could not be confirmed. The complete HPV18 genome was Sanger sequenced using 31 unique sequencing primers [15,26,27] (Table S1).

### 2.4. Phylogenetic and SNP Analysis

Sequence data were assembled against HPV18 reference strain AY262282, updated from X05015 [28] using CLC Genomics Workbench 9.5.3 (CLC Bio, Aarhus, Denmark; Qiagen, Hilden, Germany). Coverage >1 across the complete genome was required, otherwise samples were omitted. After manual verification to compensate for possible base-calling errors, consensus sequences were generated and exported to BioNumerics 7.2.5 (AppliedMaths, Sint-Martens-Latem, Belgium) for alignment and maximum parsimony phylogenetic analysis.

In addition, Multiple Sequence Comparison by Log-Expectration (MUSCLE) 3.8.31 was used to generate a multiple sequence alignment using standard settings. A maximum likelihood phylogenetic tree was generated using IQtree 1.6.1 with 1000 ultrafast bootstrap replicates (–bb 1000). An optimal substitution model was identified (–m MF) and resulted in the Kimura 3-parameter substitution model with empirical base frequencies and allowing for a proportion of invariable sites (–m K3Pu + F + I). The resulting Newick tree file was visualized using Figtree 1.4.3. Phylogenetic analyses were performed using reference strains for lineages (A, B and C) and sublineages (A1–5, B1–3) as described by [4].

SNPs were assessed using a consensus sequence generated from all sequences obtained in this study. Am SNP was considered orphan if it was found in only one participant at a single time point. Orphan SNPs were excluded from SNP analysis and comparison.

### 2.5. Statistical Analysis

General study characteristics were assessed by unpaired t or Fisher’s exact testing. Differences in SNP prevalence in clearing or persisting infections were analyzed by Fisher’s exact testing. Two-tailed *p* values < 0.05 were considered to be statistically significant. Nucleotide diversity (pi) for HPV18 clearing and persisting infections was calculated using a MUSCLE multiple alignment in DNAsp with a sliding window size of 100 nucleotides and a step size of one.

### 2.6. Accession Numbers

All HPV18 sequences were deposited to the Genbank database under accession numbers: MF288652-MF288727.

## 3. Results

**Figure 1:**
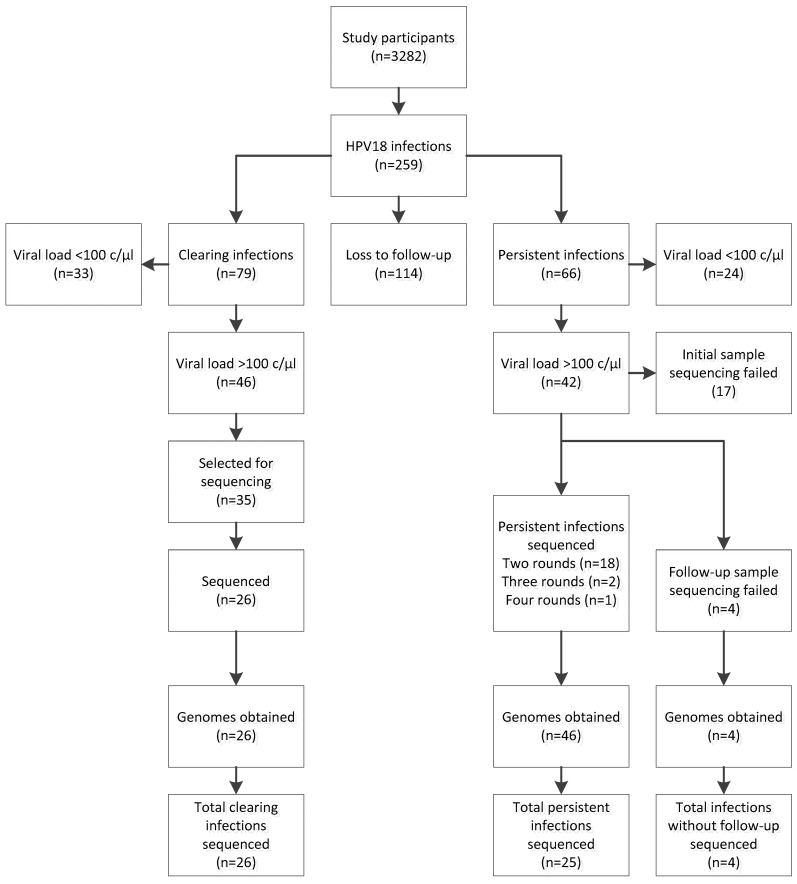
Schematic overview human papillomavirus 18 (HPV18) infections in this study, including loss to follow-up and selections made for persistent and clearing HPV18 infections.

Out of 3282 participants initially included in the CSI study, 259 were HPV18 positive (7.9%, Figure 1). Clearing and persisting infections were found in 79 (2.4%) and 66 (2.0%) study participants respectively. No follow-up information was available from 114 participants (3.5%).

Complete HPV18 genomes were obtained from 26 participants with clearing infections and 25 participants with persistent infections (Figure 1). From the 25 persistent infections, 21 were completely sequenced with at least one round of follow-up. Persistent infections had median 52.5 weeks (min 8, max 102 weeks) between sampling moments. Four persistent infections had the initial sample sequenced, but sequencing analysis failed in the follow-up. Across clearing and persisting infections combined, a total of 76 complete HPV18 sequences were obtained. General characteristics of the study subsets are presented in Table 1.

### 3.1. Phylogeny

**Figure 2:**
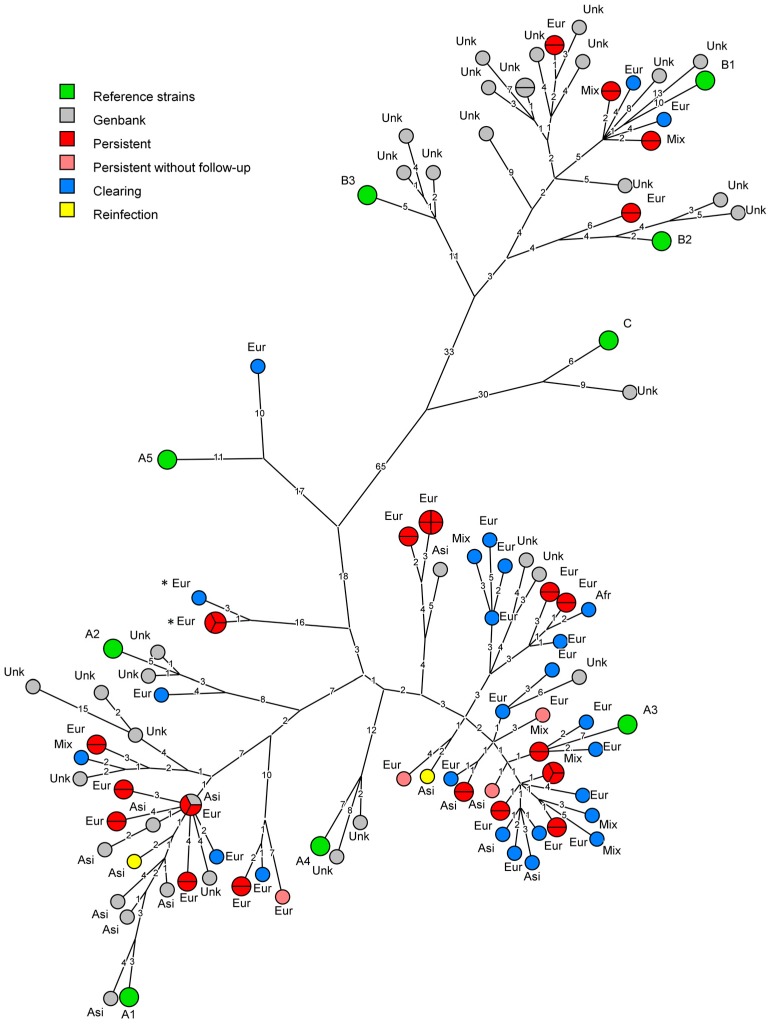
Maximum parsimony phylogenetic tree representing obtained human papillomavirus (HPV) 18 sequence data with corresponding ethnicity and compared to the currently available sequences in Genbank. Numbers on branches indicate the number of nucleotide differences between variants. Each circle represents one sequenced HPV18 variant, with the parts showing how often a variant was found. In green are reference strains for HPV18 according to [4]. Red and pink circles represent sequences obtained from persisting infections with and without sequenced follow-up respectively. Blue circles represent sequences obtained from clearing infections. The yellow circles show an infection where the initial and follow-up samples from one participant cluster differently. Grey circles represent sequences obtained from Genbank. Ethnicity for these sequences was added if available or presented as “Unk” (unknown) if not available. Marked with * are two variants who clustered poorly with any reference strain, but are still closest to A3.

A maximum parsimony phylogenetic tree of the HPV18 data was generated using BioNumerics and is shown in Figure 2. As could be expected for a Dutch cohort, most infections clustered near lineage A reference strains. A majority of the participants was infected with HPV18 sublineage A3 (64.7%, *n* = 33/51). Two participants were infected with variants clustering near the maximum allowed nucleotide difference for sublineages (0.47% and 0.49% from A3 respectively, while variants within a sublineage differ ≤0.5% at the whole genome level [4]), implying relatively large diversity can occur within a sublineage (marked with * in Figure 2 and Figure S1). Sublineage A1 was isolated and sequenced from 21.6% (*n* = 11/51) of study participants, while sublineage A2 was found only once. Lineage B HPV18 infections were also detected in this study. Five infections clustered close to sublineage B1 and one to sublineage B2. Lineage C and sublineages A4 and B3 were not found in this study. In addition, a maximum likelihood analysis was performed using MUSCLE and IQtree, which showed highly similar clustering across all (sub-) lineages (Figure S1). Both trees showed an apparent overrepresentation of clearing infections in the A3 clade (18, compared to nine persistent, excluding two sequences marked with * in Figure 2 and Figure S1) and an apparent underrepresentation of persisting infections in the A1 clade (six, compared to three clearing), however, these differences were not significant (Fisher’s exact, *p* = 0.13), even upon inclusion of persistent infections for which no follow-up could be sequenced (*p* = 0.15).

### 3.2. Analysis of Persistent Infections

Follow-up samples of 21 (out of total *n* = 25) persistent infections were sequenced. Of these infections, 18 were sequenced with one round, two with two rounds and one with three rounds of follow-up. In all women with persisting HPV18 infections, the consensus sequences found in follow-up samples were identical to those of the first sample, with one exception. This implies strong conservation of the consensus HPV18 variant within a woman for up to three years. One study participant (sampled 63 weeks apart), initially considered to have a persistent HPV18 infection, actually seemed to have an HPV18 TS reinfection, where the major variant from the initial sample clustered differently from the follow-up sample. This explains that from the 51 women studied here, we identified 52 unique HPV18 sequence variants. The consensus sequences from the apparent reinfection were verified in an independent Illumina sequencing experiment [29]. The Illumina data further showed that the variant present in the follow-up samples, was actually present in the initial samples as well, albeit at a lower ratio than the dominant variant that was identified by Sanger sequencing. The dominant variant that was identified in the initial sample was no longer present in the follow-up sample.

### 3.3. Comparison to Currently Available Data

The genomes sequenced in the current study were compared to the 47 complete HPV18 genomes described previously [14,15,16,30,31] and currently available in NCBI Genbank. The present study adds 51 new unique sequences (Figure 2). Only one sequence in the current dataset has been described previously. Overall the HPV18 whole genome data available in NCBI Genbank is more than doubled by the addition of the sequences described in this study. Especially sublineage A3 is of interest, as only five whole genome sequences have been described for this sublineage. In The Netherlands however, this turns out to be one of the dominant sublineages present in The Netherlands with 33 unique variants, resulting in a large increase of known diversity for this sublineage.

### 3.4. Ethnicity

Of all sequenced HPV18 infections in this study, 72.5% (*n* = 37/51) were found in participants of European ancestry (as proxied using country of birth). Mixed ancestry was reported by 15.7% (*n* = 8/51) of participants, Asian ancestry by 9.8% (*n* = 5/51) of participants and African ancestry by 2.0% (*n* = 1/51) of participants. Distributions of ethnicities stratified for clearing and persistent infections are shown in Table 1 and Figure 2. The distribution of ethnicities of the infections sequenced here was not significantly different from the complete study for any of the represented ethnicities. For this dataset, sequences obtained from participants with non-European ancestry were limited. However, no sublineages seemed to occur preferentially in specific ethnic groups.

### 3.5. SNP Analysis

**Figure 3:**
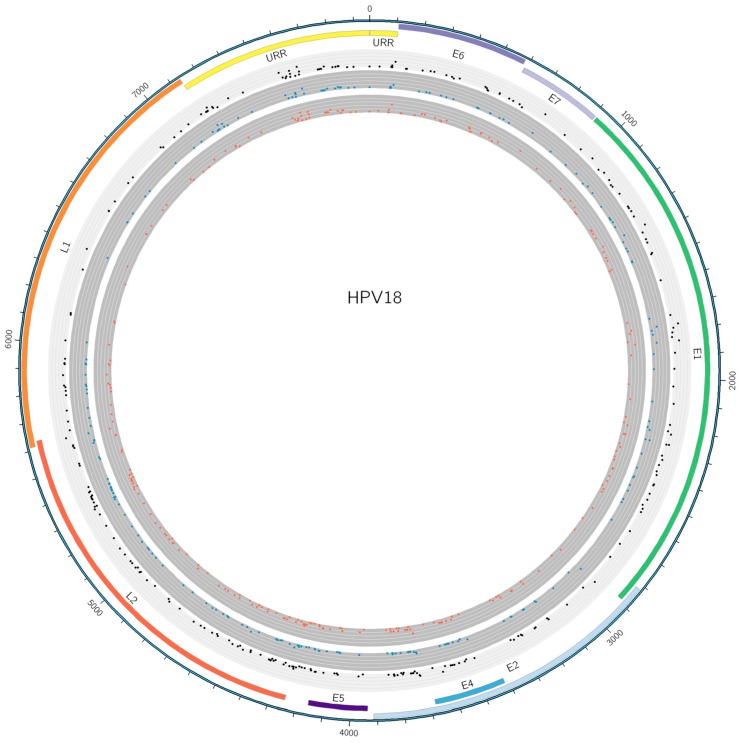
Circular plot of non-orphan single-nucleotide polymorphisms (SNPs) on the human papillomavirus (HPV) 18 genome. Each dot denotes a variable site within the dataset. Black dots show total SNPs occurring in this study. Blue dots were variations identified in clearing infections, while reds dots were variations identified in persisting infections. Total DNA variations are shown on the light grey circles. Height of dots on the respective circles shows how often the variation occurs in the dataset.

From the 52 unique HPV18 variants sequenced in this study, 334 SNPs were identified. After exclusion of orphan SNPs not observed in any other participant or follow-up sample, 244 SNPs remained, a large number of which have not been described previously (Figure 3, Table S1). Furthermore, six deletions were found across the various variants. None of the sequences included in this study contained any insertions. Of the 244 non-orphan SNPs, 85 caused amino-acid (AA) changes (Table S2). Three out of six deletion events were found in coding regions of the genome (12 bp E1, 6 bp E2/E4 and 15 bp L2). None of the found deletions caused frameshifts or AA changes, including the deletion found in the shared open-reading frame (ORF) for E2 and E4. The other three deletions were identified in the intergenic region between E2 and E5 ORFs (20 bp) and the upstream regulatory region (URR) (26 bp and 7 bp). The E2 deletion and the 7 bp URR were linked for this study. Six participants were found with both deletions and none with either one of the deletions. In addition five of these six study participants also had the 20 bp deletion between the E2 and E5 ORFs, suggesting another link, albeit not exclusive. This deletion was not identified on its own in any of the other study participants. The 12 bp E1, 15 bp L2 and 26 bp URR deletions have not been described previously, although they were only identified in one study participant each.

**Figure 4:**
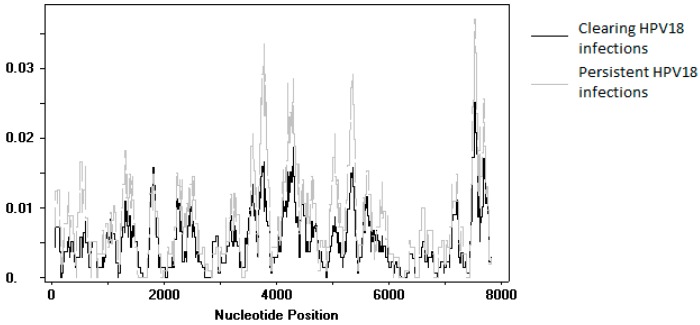
Plot of nucleotide diversity (pi) of human papillomavirus (HPV) 18 sequences obtained in this study. The black line shows data from clearing infections (*n* = 25), while the grey line shows data from persistent infections (*n* = 20).

An SNP comparison was made between sequences obtained from persisting and clearing HPV18 infections. Upon exclusion of the variants identified in the reinfection event and the variants for which the follow-up samples could not be sequenced, SNPs were compared between 20 persistent infections and 26 clearing infections. No individual SNP was found to be significantly different between the two groups. A sliding window analysis of genome diversity showed no large differences between the two groups (Figure 4).

## 4. Discussion

In this study, we describe the remarkably large diversity of HPV18 variants circulating in a Dutch cohort study based on whole genome sequencing data. Due to one HPV18 infection with two different consensus variants at follow-up sampling moments, we actually identified 52 unique variants from 51 study participants. Despite this diverse population in study participants, we find that in persistent infections, the same consensus sequence remains completely conserved in up to three years of follow-up.

With this study, the number of published complete HPV18 genome sequences has been doubled. Of the 52 unique sequences found in our study, 51 are unique in the NCBI Genbank database for whole genome HPV18 sequences. To date only three studies have described and compared multiple HPV18 whole genome sequences, highlighting the necessity of HPV18 population studies. These three studies have included samples from South-America, Africa and Southeast Asia. European studies are scarce and to our knowledge we are the first to describe whole genome HPV18 diversity for a European country. Interestingly, phylogenetic analysis shows the variant diversity partially matches the variants described to date, but in this study there is a heavy emphasis on sublineage A3 variants, which is underrepresented in other studies. This suggests that in The Netherlands, and possibly Europe, there is a different distribution of sublineages than in other parts of the world. In both phylogenetic analyses performed in this study, persisting and clearing infections seem to be distributed differently in the A1 and A3 clades, however, the distribution of clearing and persistent infections between these clades was not statistically significant. This may be due to the relatively small sample size and it would be of interest to see if these differences in distribution also occur in larger datasets.

It should be noted that during initial amplification, seventeen persisting and nine clearing infections failed to amplify. A further four persistent infections were successfully sequenced in the initial round, but failed in the follow-up sample. This might suggest a PCR bias in the sequenced group and a possible underrepresentation of variants that could not be amplified by our PCR assay. This explanation seems unlikely because the primers used for this study, as they were designed against conserved areas of the HPV18 genome and variants from six different sublineages were successfully amplified. 

Considering the single occurrence of an HPV18 infection with two different major variants in follow-up samples that we found from 21 study participants with persistent HPV18 infections, we can conclude that conventional genotyping leads to reliable identification of persistent infections. However, in the context of vaccine studies, a single persistent infection could affect measured vaccine efficacy or efficiency in a negative manner, while this might actually be a repeat incident infection after sequencing. In such specific cases, it could be warranted to confirm if it is indeed a persistent infection, or actually a type-specific reinfection. In fact, the next-generation sequencing (NGS) confirmation of apparent reinfection [29], suggested that at the initial sampling moment, the variant from the follow-up sample was already present at a low ratio. Upon reinspection of the Sanger data, secondary peaks could be distinguished at low signal for the variable sites identified by NGS. This suggests that this infection meets both the criteria for a persisting (HPV18 positive with HPV18 positive follow-up) and a clearing infection (HPV18 positive, with HPV18 negative follow-up), based on the variant analyzed. In the follow-up sample, the initial variant was not identified from the NGS experiment. This suggests that our Sanger sequencing results were reliable in assessing persistent infections where the same consensus sequence is identified in follow-up samples, but not in the case of reinfections, where a cocktail of variants might be present and variant-specific clearance may occur.

The data obtained in this study identifies a large number of new SNPs. In addition, six deletions were found, of which the coding one on E2/E4 and the non-coding ones on the E2 E5 intergenic region and the URR (7 bp) have been described previously [14,15,16]. Three other deletions which have not been described previously were identified once each in this study population. This could suggest a possible artifact; however, the deletions on E1 and the URR (26 bp) were identified from persistent infections. The same deletions were found in three rounds for the E1 deletion and in two rounds for the 26 bp URR deletions which strongly argues for true signs of diversity and not diagnostic artifacts. The deletion found in L2 was only identified once from a clearing infection. This deletion, along with orphan SNPs identified only once in this dataset, could not be confirmed due to scarcity of the material and therefore we cannot exclude that these represent possible PCR artifacts.

The genomes described in this study were obtained through Sanger sequencing. In the current era, next-generation sequencing is rapidly emerging as the go-to method for sequencing. It generates far more data at a higher sensitivity than Sanger sequencing, thereby allowing for the identification of intra-patient HPV co-infections and even HPV type-specific variant co-infections [9]. Such findings are beyond the resolution of Sanger sequencing. Despite this, Sanger remains the golden standard in sequencing and, for our rationale, an adequate, cheap technique to use. The robustness of Sanger sequencing is shown in this study by the consistent identification of the same consensus sequence through time in persistent infections.

In addition, we have attempted to compare sequences obtained from persistent and clearing infections. For HPV16, it has been shown that E7 conservation is higher in infections that eventually progress to CIN3+ [10]. In this study, we do observe strong conservation of E7 for HPV18, but the number of infections sequenced in this study is too small to identify any differences between sequences obtained from persistent or clearing infections. To gain insight into the role of the HPV18 genome on infection duration, large-scale NGS studies should be performed.

Previous studies have been ambiguous in describing an association between HPV18 (sub)lineages and specific ethnic groups with regards to persistent infections and the development of cervical cancer [8,27,32,33]. The dataset in this study is too limited in size to identify clear links between the occurrence of (persistent) HPV18 variants in population subgroups. 

Despite the limited study size, we have shown the natural occurrence of a large pool of HPV18 variants in a Dutch population of young women. Our findings stress the importance of identifying the different properties associated with various variants and (sub)lineages for HPV18. When comparing clearing and persistent infections, no specific SNPs were identified as strongly affecting the outcome of infection, although the size of the dataset used here could only identify very strong-acting effects.

## Figures and Tables

**Table 1:** General characteristics of study participants with human papillomavirus 18 positive results. Subsets were not found to be significantly different from the complete study population with regard to age, C. trachomatis status and ethnicity (*p* > 0.05).

General Subset Statistics	Infections (*n* Sequenced/*n* Total)	
Persistent	25/66	
Clearing	26/79	
Age	Median years (95% CI)	
	Persistent	Clearing
Total dataset	24 (23–25)	25 (23–26)
Sequenced subset	24 (21–25)	23 (20–26)
Unpaired *t*-test	*p* = 0.28	*p* = 0.89
*C. trachomatis* status	*C. trachomatis* positive (*n*)/Total (*n*)	
	Persistent	Clearing
Total dataset	6/66	8/79
Sequenced subset	3/25	3/26
Fisher’s exact test	*p* = 0.70	*p* = 1
European	European (*n*)/Total (*n*)	
	Persistent	Clearing
Total dataset	51/66	55/79
Sequenced subset	18/25	19/26
Fisher’s exact test	*p* = 0.59	*p* = 0.81
Mixed	Mixed (*n*)/Total (*n*)	
	Persistent	Clearing
Total dataset	8/66	12/79
Sequenced subset	4/25	4/26
Fisher’s exact test	*p* = 0.73	*p* = 1.0
Asian	Asian (*n*)/Total (*n*)	
	Persistent	Clearing
Total dataset	6/66	8/79
Sequenced subset	3/25	2/26
Fisher’s exact test	*p* = 0.70	*p* = 1.0
African	African (*n*)/Total (*n*)	
	Persistent	Clearing
Total dataset	0/66	3/79
Sequenced subset	0/25	1/26
Fisher’s exact test	*p* = 1.0	*p* = 1.0

## Supplementary Materials

The following are available online at http://www.mdpi.com/1999-4915/10/2/68/s1, Figure S1: Maximum likelihood tree of sequences obtained in this study, Table S1: Nucleotide alignment of sequences obtained in this study, aligned to reference strain AY262282, Table S2: Amino-acid alignment of sequences obtained in this study, aligned to reference strain AY262282.

## References

[1] Walboomers J.M., Jacobs M.V., Manos M.M., Bosch F.X., Kummer J.A., Shah K.V., Snijders P.J., Peto J., Meijer C.J., Munoz N. (1999). Human papillomavirus is a necessary cause of invasive cervical cancer worldwide. J. Pathol..

[2] IARC Working Group on the Evaluation of Carcinogenic Risks to Humans (2012). Biological agents. Volume 100 B. A review of human carcinogens. IARC Monogr. Eval. Carcinog. Risks Hum..

[3] De Sanjose S., Quint W.G., Alemany L., Geraets D.T., Klaustermeier J.E., Lloveras B., Tous S., Felix A., Bravo L.E., Shin H.R. (2010). Human papillomavirus genotype attribution in invasive cervical cancer: A retrospective cross-sectional worldwide study. Lancet Oncol..

[4] Burk R.D., Harari A., Chen Z. (2013). Human papillomavirus genome variants. Virology.

[5] Xi L.F., Koutsky L.A., Hildesheim A., Galloway D.A., Wheeler C.M., Winer R.L., Ho J., Kiviat N.B. (2007). Risk for high-grade cervical intraepithelial neoplasia associated with variants of human papillomavirus types 16 and 18. Cancer Epidemiol. Biomark. Prev..

[6] Xi L.F., Schiffman M., Koutsky L.A., Hughes J.P., Winer R.L., Mao C., Hulbert A., Lee S.K., Shen Z., Kiviat N.B. (2014). Lineages of oncogenic human papillomavirus types other than type 16 and 18 and risk for cervical intraepithelial neoplasia. J. Natl. Cancer Inst..

[7] Mirabello L., Yeager M., Cullen M., Boland J.F., Chen Z., Wentzensen N., Zhang X., Yu K., Yang Q., Mitchell J. (2016). HPV16 Sublineage Associations With Histology-Specific Cancer Risk Using HPV Whole-Genome Sequences in 3200 Women. J. Natl. Cancer Inst..

[8] Chen A.A., Gheit T., Franceschi S., Tommasino M., Clifford G.M. (2015). Human Papillomavirus 18 Genetic Variation and Cervical Cancer Risk Worldwide. J. Virol..

[9] Cullen M., Boland J.F., Schiffman M., Zhang X., Wentzensen N., Yang Q., Chen Z., Yu K., Mitchell J., Roberson D. (2015). Deep sequencing of HPV16 genomes: A new high-throughput tool for exploring the carcinogenicity and natural history of HPV16 infection. Papillomavirus Res..

[10] Mirabello L., Yeager M., Yu K., Clifford G.M., Xiao Y., Zhu B., Cullen M., Boland J.F., Wentzensen N., Nelson C.W. (2017). HPV16 E7 Genetic Conservation Is Critical to Carcinogenesis. Cell.

[11] Xi L.F., Schiffman M., Koutsky L.A., Hughes J.P., Hulbert A., Shen Z., Galloway D.A., Kiviat N.B. (2016). Variant-specific persistence of infections with human papillomavirus Types 31, 33, 45, 56 and 58 and risk of cervical intraepithelial neoplasia. Int. J. Cancer.

[12] Van Belkum A., Juffermans L., Schrauwen L., van Doornum G., Burger M., Quint W. (1995). Genotyping human papillomavirus type 16 isolates from persistently infected promiscuous individuals and cervical neoplasia patients. J. Clin. Microbiol..

[13] Van der Weele P., Meijer C., King A.J. (2017). Whole-Genome Sequencing and Variant Analysis of Hpv16 Infections. J. Virol..

[14] Chen Z., DeSalle R., Schiffman M., Herrero R., Burk R.D. (2009). Evolutionary dynamics of variant genomes of human papillomavirus types 18, 45, and 97. J. Virol..

[15] Lurchachaiwong W., Junyangdikul P., Termrungruanglert W., Payungporn S., Sampatanukul P., Tresukosol D., Niruthisard S., Trivijitsilp P., Karalak A., Swangvaree S. (2010). Whole-genome sequence analysis of human papillomavirus type 18 from infected Thai women. Intervirology.

[16] Chen Z., Schiffman M., Herrero R., DeSalle R., Anastos K., Segondy M., Sahasrabuddhe V.V., Gravitt P.E., Hsing A.W., Burk R.D. (2013). Evolution and taxonomic classification of alphapapillomavirus 7 complete genomes: HPV18, HPV39, HPV45, HPV59, HPV68 and HPV70. PLoS ONE.

[17] Apter D., Wheeler C.M., Paavonen J., Castellsague X., Garland S.M., Skinner S.R., Naud P., Salmeron J., Chow S.N., Kitchener H.C. (2015). Efficacy of human papillomavirus 16 and 18 (HPV-16/18) AS04-adjuvanted vaccine against cervical infection and precancer in young women: Final event-driven analysis of the randomized, double-blind PATRICIA trial. Clin. Vaccine Immunol..

[18] Joura E.A., Giuliano A.R., Iversen O.E., Bouchard C., Mao C., Mehlsen J., Moreira E.D., Ngan Y., Petersen L.K., Lazcano-Ponce E. (2015). A 9-valent HPV vaccine against infection and intraepithelial neoplasia in women. N. Engl. J. Med..

[19] Van den Broek I.V., Hoebe C.J., van Bergen J.E., Brouwers E.E., de Feijter E.M., Fennema J.S., Gotz H.M., Koekenbier R.H., van Ravesteijn S.M., de Coul E.L. (2010). Evaluation design of a systematic, selective, internet-based, Chlamydia screening implementation in The Netherlands, 2008–2010: Implications of first results for the analysis. BMC Infect. Dis..

[20] Van den Broek I.V., van Bergen J.E., Brouwers E.E., Fennema J.S., Gotz H.M., Hoebe C.J., Koekenbier R.H., Kretzschmar M., Over E.A., Schmid B.V. (2012). Effectiveness of yearly, register based screening for chlamydia in The Netherlands: Controlled trial with randomised stepped wedge implementation. BMJ.

[21] Mollers M., Boot Hein J., Vriend Henrike J., King Audrey J., van den Broek Ingrid V.F., van Bergen Jan E.A., Brink Antoinette A.T., Wolffs Petra F.G., Hoebe Christian J.P., Meijer Chris J.L. (2013). Prevalence, incidence and persistence of genital HPV infections in a large cohort of sexually active young women in The Netherlands. Vaccine.

[22] Woestenberg P.J., van Oeffelen A.A., Stirbu-Wagner I., van Benthem B.H., van Bergen J.E., van den Broek I.V. (2015). Comparison of STI-related consultations among ethnic groups in The Netherlands: An epidemiologic study using electronic records from general practices. BMC Fam. Pract..

[23] Kleter B., van Doorn L.J., Schrauwen L., Molijn A., Sastrowijoto S., ter Schegget J., Lindeman J., ter Harmsel B., Burger M., Quint W. (1999). Development and clinical evaluation of a highly sensitive PCR-reverse hybridization line probe assay for detection and identification of anogenital human papillomavirus. J. Clin. Microbiol..

[24] Kleter B., van Doorn L.J., ter Schegget J., Schrauwen L., van Krimpen K., Burger M., ter Harmsel B., Quint W. (1998). Novel short-fragment PCR assay for highly sensitive broad-spectrum detection of anogenital human papillomaviruses. Am. J. Pathol..

[25] Van der Weele P., van Logchem E., Wolffs P., van den Broek I., Feltkamp M., de Melker H., Meijer C.J., Boot H., King A.J. (2016). Correlation between viral load, multiplicity of infection, and persistence of HPV16 and HPV18 infection in a Dutch cohort of young women. J. Clin. Virol..

[26] Arroyo S.L., Basaras M., Arrese E., Hernaez S., Andia D., Esteban V., Garcia-Etxebarria K., Jugo B.M., Cisterna R. (2012). Human papillomavirus (HPV) genotype 18 variants in patients with clinical manifestations of HPV related infections in Bilbao, Spain. Virol. J..

[27] King A.J., Sonsma J.A., Vriend H.J., van der Sande M.A., Feltkamp M.C., Boot H.J., Koopmans M.P., Medical Microbiological L., Municipal Health S. (2016). Genetic Diversity in the Major Capsid L1 Protein of HPV-16 and HPV-18 in The Netherlands. PLoS ONE.

[28] Cole S.T., Danos O. (1987). Nucleotide sequence and comparative analysis of the human papillomavirus type 18 genome. Phylogeny of papillomaviruses and repeated structure of the E6 and E7 gene products. J. Mol. Biol..

[29] Van der Weele P. (2016). National Institute for Public Health and the Environment (RIVM).

[30] Siqueira J.D., Alves B.M., Prellwitz I.M., Furtado C., Meyrelles A.R., Machado E.S., Seuanez H.N., Soares M.A., Soares E.A. (2016). Identification of novel human papillomavirus lineages and sublineages in HIV/HPV-coinfected pregnant women by next-generation sequencing. Virology.

[31] Oliveira G.R., Siqueira J.D., Finger-Jardim F., Vieira V.C., Silva R.L., Goncalves C.V., Soares E.A., Martinez A.M.B., Soares M.A. (2017). Characterisation of complete high- and low-risk human papillomavirus genomes isolated from cervical specimens in southern Brazil. Memórias do Instituto Oswaldo Cruz.

[32] Xi L.F., Kiviat N.B., Hildesheim A., Galloway D.A., Wheeler C.M., Ho J., Koutsky L.A. (2006). Human papillomavirus type 16 and 18 variants: Race-related distribution and persistence. J. Natl. Cancer Inst..

[33] Lizano M., de la Cruz-Hernandez E., Carrillo-Garcia A., Garcia-Carranca A., Ponce de Leon-Rosales S., Duenas-Gonzalez A., Hernandez-Hernandez D.M., Mohar A. (2006). Distribution of HPV16 and 18 intratypic variants in normal cytology, intraepithelial lesions, and cervical cancer in a Mexican population. Gynecol. Oncol..

