# Supplementary material for: High Whole-Genome Sequence Diversity of Human Papillomavirus Type 18 Isolates
Source: Viruses. 2018 Feb 7;10(2):68. doi: 10.3390/v10020068 (PMC5850375; doi:10.3390/v10020068)
Supplement: Supplementary file 1 [file viruses-10-00068-s001.zip › supplemenraty-revised/Captions Supplementary revised.pdf]

Figure S1: Maximum likelihood phylogenetic tree representing obtained HPV18 sequence data with corresponding ethnicity and compared to the currently available sequences in Genbank. Numbers on the nodes indicate bootstrapping support based on 1000 ultrafast bootstraps using the IQtree ultrafast bootstrapping algorithm. CSI samples were class-encoded using P for persistent infections, C for clearing infections, PNF for persistent infection where follow-up could not be sequenced and R for the reinfection event. The first number following class is infection number, followed by the follow-up sample and the accession number. Persistent infections with identical variants in follow-up samples were collapsed into a single entry. In green are reference strains for HPV18 according to [4]. Red and pink names represent sequences obtained from persisting infections with and without sequenced follow-up respectively. Blue names represent sequences obtained from clearing infections. The yellow names show an infection where the initial and follow-up samples from one participant cluster differently. Black names represent sequences obtained from Genbank. Ethnicity for these sequences was added if available or presented as “Unk” (unknown) if not available. Marked with * are two variants who clustered poorly with any reference strain, but are still closest to A3.

Table S1: Nucleotide alignment of sequences obtained in this study, aligned to reference strain AY262282. N-P (non-persistent) sequences were obtained from clearing infections. Persistent infections have the duration in weeks displayed after the sample number. Genes with amino-acid ORFs are displayed on the far right. Nucleotide positions that differ from the consensus are displayed on the far-right column, along with the variation being either a single-nucleotide polymorphism (SNP) or deletion (DEL).

Table S2: Amino-acid alignment of sequences obtained in this study, aligned to reference strain AY262282. N-P (non-persistent) sequences were obtained from clearing infections. Persistent infections have the duration in weeks displayed after the sample number. Amino-acid positions that differ from the consensus are displayed on the far-right column.
