# Supplementary figures and images for: High Whole-Genome Sequence Diversity of Human Papillomavirus Type 18 Isolates
Source: Viruses. 2018 Feb 7;10(2):68. doi: 10.3390/v10020068 (PMC5850375; doi:10.3390/v10020068)

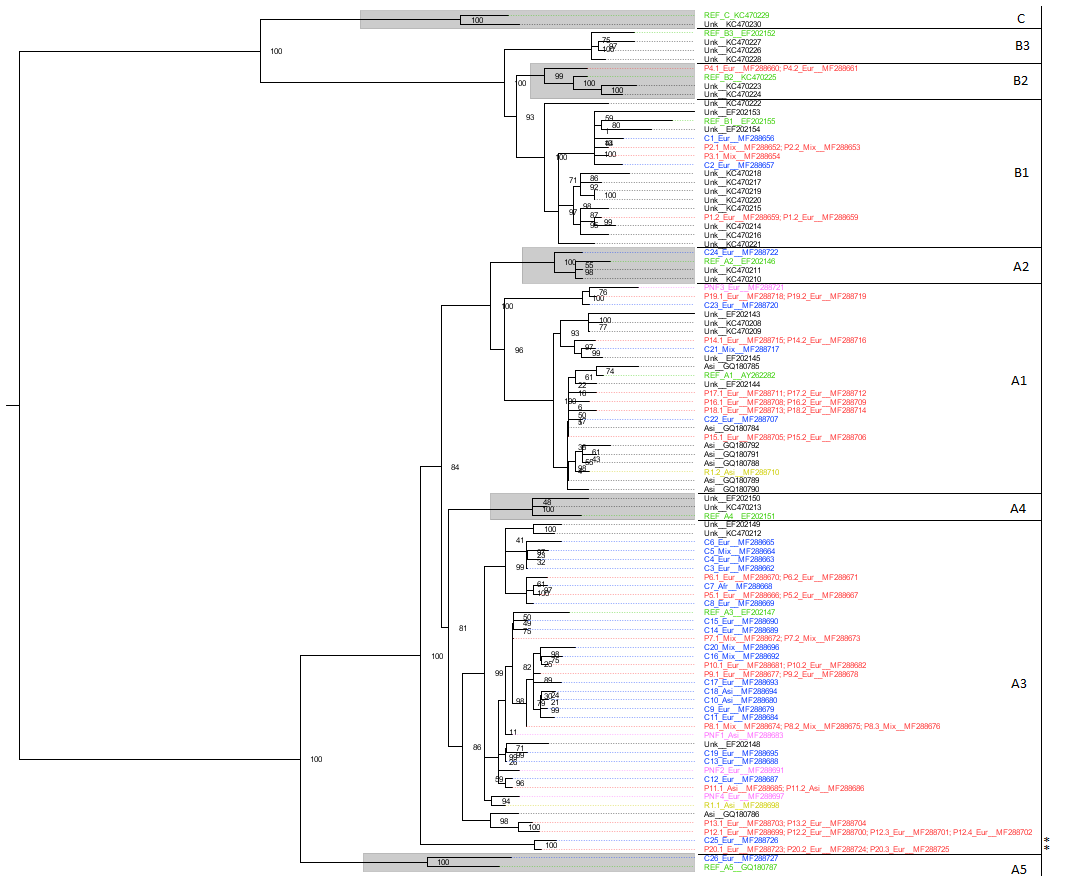

Supplement: Supplementary file 1 [file viruses-10-00068-s001.zip › supplemenraty-revised/Figure S1 revised 20180202.png]
